# The Last Phase of Quackery

**Published:** 1856-04

**Authors:** 


*The Last Phase of Quackery.*—The remarkable fact has been discovered in Memphis, hitherto unknown to philosophers and telegraphists, that the electro-magnetism may be generated by a newly-invented black plaster, the composition of which is a profound secret; and that tertian and quartan agues can be cured by its electrical influence in *four hours.* We have not heard of its being applied to telegraphic purposes; but of course it will be, and perhaps become, also, an important agent of motive power, as soon as its composition is made known. Like infinitesimalism, it is commended as an entirely harmless medical agent. The only injury to be apprehended from its use is, that it may supersede other remedial measures, until it is too late to employ them for the relief of the patient. That it is a sovereign remedy for "chills and fevers," and destined to "drive quinine from its throne," we have the assurance of the inventor; and he is fully sustained by editors of newspapers, and certain other persons, all excellent judges of disease, and the proper treatment thereof.—*Memphis Med. Recorder.*

